# Ghosts of Cultivation Past - Native American Dispersal Legacy Persists in Tree Distribution

**DOI:** 10.1371/journal.pone.0150707

**Published:** 2016-03-16

**Authors:** Robert J. Warren

**Affiliations:** Department of Biology, SUNY Buffalo State, 1300 Elmwood Avenue, Buffalo, NY, 14222, United States of America; University of Toronto Mississauga, CANADA

## Abstract

A long-term assumption in ecology is that species distributions correspond with their niche requirements, but evidence that species can persist in unsuitable habitat for centuries undermines the link between species and habitat. Moreover, species may be more dependent on mutualist partners than specific habitats. Most evidence connecting indigenous cultures with plant dispersal is anecdotal, but historical records suggest that Native Americans transported and cultivated many species, including *Gleditsia triacanthos* ("Honey locust"). *Gleditsia triacanthos* was an important medicinal/culinary (e.g., sugar), cultural (e.g., game sticks) and spiritual tree for the Cherokee (southeastern U.S. Native Americans). This study tests the hypothesis that a Cherokee cultivation legacy drives current regional *G*. *triacanthos* distribution patterns. *Gleditsia triacanthos* occurs in rocky uplands and xeric fields, but inexplicably also occurs in mesic riverine corridors and floodplains where Cherokee once settled and farmed. I combined field experiments and surveys in the Southern Appalachian Mountain region (U.S.) to investigate *G*. *triacanthos* recruitment requirements and distribution patterns to determine whether there is a quantifiable *G*. *triacanthos* association with former Cherokee settlements. Moreover, I also investigated alternate dispersal mechanisms, such as stream transport and domestic cattle. The results indicate that a centuries-old legacy of Native American cultivation remains intact as *G*. *triacanthos*' current southern Appalachian distribution appears better explained Cherokee settlement patterns than habitat. The data indicate that the tree is severely dispersal limited in the region, only moving appreciable distances from former Cherokee settlements where cattle grazing is prevalent. Human land use legacy may play a long-term role in shaping species distributions, and pre-European settlement activity appears underrated as a factor influencing modern tree species distributions.

## Introduction

A simplifying assumption in ecology and biogeography is that species occupy suitable habitat and are absent from unsuitable habitat. The veracity of that assumption is undermined, however, by evidence that species persist in unsuitable habitat [1, 2] and remain absent from suitable habitat [3, 4]. Plants that depend on a mutualist may be strongly associated with habitat suitable for their partner [5–7] so that some plant distributions better reflect the niche requirements of the mutualist than the plant itself. Conversely, the absence (or loss) of a dispersal partner reduces (or eliminates) the plant’s ability to track suitable habitat and may leave it stranded in suboptimal habitat [2]. As such, plant populations can persist in less than optimal conditions for centuries [8] and may be in disequilibrium with optimal habitat at very large scales [9].

The ecological legacy of European plant migrants in North America is well studied ([10]); much less is known about the ecological impacts of indigenous populations. Early explorers noted a relationship between Native American land use and specific plant species [11–13], such as the proximity between fruit and nut trees and indigenous trade routes and settlements [14, 15]. In many cases, former Native American settlements contain plant populations that are separate from their main, contiguous distributions, suggesting that the plants were moved and planted; however, linking disjunct plant distributions with Native American cultivation remains a contentious and unresolved matter [15–18]: Native Americans may have created habitat that favors specific species (e.g., with fire and farming) or they may have shared habitat preferences with those species (e.g., riverine bottomlands) without actively transporting and cultivating them (see [11, 16, 19]). *Gleditsia triacanthos* (“honey locust”) trees often occur in rocky uplands and xeric fields in the western portion of its main range, but also occur in wet bottomlands and floodplains in the eastern portion [20–23], the same habitats where Cherokee travelled, settled and farmed [19, 24].

The Cherokee occupied at least 322,000 km^2^ of the Southern Appalachian region in a fragmented, loose-knit settlement pattern with towns and farms located along rivers and streams in flatlands and mountain valleys (see [19, 25]). The Cherokee used *G*. *triacanthos* in the southeastern U.S for food, medicine, weapons and game sticks [26–29]. “Kulsetsiyi” is a Cherokee name for “honey locust place” (rendered “Cullasaja” or “sugartown” by European traders) that was used for Cherokee settlements reportedly containing honey locust orchards (Mooney, 1900). Moreover, in the 1700s, both Bartram (12) and Lawson (13) noted *G*. *triacanthos* growing near eastern U.S. Native American settlements.

An excellent opportunity to investigate the link between Native Americans and cultivated trees is the occurrence of *G*. *triacanthos* in the southern Appalachian Mountain region (U.S.) as the landscape remains largely rural with many former Cherokee settlement locations free of intensive development. Moreover, *G*. *triacanthos* dispersal likely coevolved with now extinct Pleistocene megafauna [16, 30], and current *G*. *triacanthos* populations appear severely dispersal limited in the U.S. [23], so that the legacy of historical Cherokee cultivation may persist in the current landscape.

My overall objective was to examine whether *G*. *triacanthos*' Southern Appalachian Mountain distribution was shaped by a legacy of Cherokee planting. Given that *G*. *triacanthos* exhibits broad-scale distribution patterns that occur in both xeric and mesic conditions, always in open habitats, I tested whether the southern Appalachian Mountain region contains suitable habitat using germination experiments and seed addition experiments in the field. Seed introduction experiments are the standard method for decoupling seed and establishment limitations [31, 32], and they are especially effective if introduced to heterogeneous microsites [3]. I then conducted a series of extensive field surveys to examine associations between *G*. *triacanthos* trees and Cherokee settlement sites. I hypothesized that *G*. *triacanthos* distributions in the region are best predicted by a Cherokee cultivation legacy.

My secondary objective was to examine alternative dispersal mechanisms, if any, for *G*. *triacanthos*. One explanation for the association between riverine corridors and tree species with extinct dispersers is that water transport provides a viable dispersal mechanism [15, 16]. An additional possibility is that other animals currently disperse the seeds [16, 21, 33], specifically domestic cattle [21, 30, 33].

## Materials and Methods

### Study Species and Site Permissions

*Gleditsia triacanthos* L. is a leguminous tree that produces long (15–40 cm), flattened seed pods filled with ~30 hard-coated seeds within a starchy, sweet fibrous matrix. The tree’s range stretches from the Great Lakes to the Gulf Coast in the middle of North America, but the eastern limit ends approximately 80 km from the study region [22, 34]. Surveys and field experiments were conducted on public and private lands throughout the Southern Appalachian Mountain region. Sampling procedures and experimental manipulations were reviewed and permissions granted by the Eastern Band of Cherokee Indians (ECBI) Tribal Historic Preservation Office, the Land Trust of the Little Tennessee, and the Great Smoky Mountains National Park under permit GRSM-2014-SCI-1170. As the survey work was non-destructive, permission was not required to survey trees on the other public sites, which generally were public parks and right-of-ways. Permission was secured directly from private landowners where needed.

### Recruitment Experiment—Germination

Fruits were collected from two *G*. *triacanthos* trees at the Tessentee Bottomland Preserve (35°04’03.57”N; 83°23’00.53”W) near Franklin, NC (U.S.). Seeds were extracted from the fruit pulp, and any seeds that were poorly formed or damaged by beetle larvae were removed. The remaining seeds were mixed and stored at 4°C for 6 months. In April 2009, the seeds were soaked in 90% concentrated sulfuric acid for two hours and rinsed thoroughly with water. The scarification of seeds is consistent with megafaunal dispersal (including cattle and horses) as the large seed pods pass through the digestive system, or Native American cultivation that including soaking or boiling the pods [30, 35, 36].

Scarified seeds were placed in a peat medium, randomly placed in 12 covered germination trays and exposed to 100% ambient sunlight (*n* = 36), 50% ambient sunlight (*n* = 36), 100% soil moisture saturation (*n* = 36) and 50% soil moisture saturation (*n* = 36). Ambient sunlight was ameliorated using 50% shadecloth (International Greenhouse Co., Georgetown, Illinois, U.S.). Light measurements were taken five times during the germination experiment with a LiCor LI-191 Line Quantum Sensor (LiCor Biosciences, Lincoln, NE, U.S.). Soil moisture saturation was controlled by adding water until the peat medium was 100% saturated without standing water in undrained pots. For 50% saturation, 50% the amount of water required for saturation was added, and the container bottoms were perforated to allow drainage.

### Recruitment Experiment—Seed Addition

I experimentally planted *G*. *triacanthos* seeds near the Little Tennessee River in Macon County, North Carolina (U.S.). Historically, the floodplains and terraces of the Little Tennessee Valley were extensively settled by Cherokee who built settlements and ceremonial mounds and farmed in the area [28, 29]. A major Cherokee settlement, Nikwasi, is located near the confluence of the Little Tennessee and Cullasaja Rivers, and this confluence was reported as containing one of three southeastern U.S. *G*. *triacanthos* orchards [28]. Both of the recruitment field sites contained historical Cherokee settlements and are managed by the Little Tennessee Land Trust. The first study site, Gibson Bottoms, is a 15-ha tract (elev: 600–610 m); the second site, Tessentee Bottomland Preserve, is a 26-ha tract (elev: 612–622 m). Preliminary searches at both sites indicated a high degree of seed production by a few adult *G*. *triacanthos* trees, but little seed recruitment. This observation prompted the seed addition experiments to examine whether recruitment was habitat limited. Each site contains mixed open (fields) and closed (early to mid-successional forest) habitats, and the riverine geomorphology includes riparian terraces and wet floodplain depressions. Three habitat types—forest edge, floodplain and riparian—were chosen to expose seeds to soil moisture, temperature and light gradients.

In May 2009, scarified *G*. *triacanthos* seeds were planted 3 cm deep at both sites (Tessentee and Gibson Bottoms) and in each habitat type (forest edge, floodplain, riparian terrace). Five seeds were planted (*n* = 126 plots, 630 seeds) at 5-m intervals along transects that paralleled the Little Tennessee River at distances ranging from approximately 10 m (riparian), 20 m (floodplain) and 30 m (forest edge). Germination success was measured as the proportion of seeds that sprouted within 30 days. The plots were re-surveyed in September 2009 and 2011 to assess seedling survival.

Volumetric soil moisture (%) was measured at five averaged points within 12 cm of each plot with a handheld Hydrosense Soil Water Content Measurement System (Campbell Scientific, Inc., Logan, Utah, U.S.). Percent PPFD (diffuse light) was calculated as the difference between plot-level (0.25 m above ground) PPFD readings and a fully exposed PPFD reference site. The plot-level measurements were taken with the LI-191 line quantum sensor and the open reference measurements were taken with an LI-200 spherical PAR sensor at 0.25 m height and logged with a LI-1400 datalogger (LiCor, Inc., Lincoln, Nebraska, U.S.). Measurements were taken on cloudy days between 10 a.m. and 2 p.m. to minimize relative error in diffuse light. Ambient soil temperature was measured in each plot with a T-shaped digital thermometer inserted 8 cm into the ground. The amount (%) of area covered by existing plants was estimated for the area (1 m^2^) surrounding each plot and reported as vegetation cover. Temperature and soil moisture data were collected in April and September 2009 and 2011, and mean values were used for analysis.

### Cherokee Settlement Sites

All of the settlement sites surveyed here were occupied during the Qualla phase of the Cherokee culture (A.D. 1450–1838). The Qualla phase of southern Appalachian Mountain region Cherokee was marked by changes in pottery from incised to stamped markings, town houses as focal points of local clans and subsistence farming in river bottomlands [12, 37–40]. Many settlement areas also featured earthen platform mounds that were built before the Qualla phase, but used for religious and political ceremonies until the towns were abandoned after European contact [12, 28, 41]. Many of the town names are not Cherokee, and may have been established by other tribes, most likely the Muscogee (Creek) Indians that once inhabited most of the Southeastern U.S. [42]. The Cherokee towns loosely grouped into five major divisions [37, 43]–the Middle, Valley and Overhill town areas occurred in the Southern Appalachian Mountain region surveyed here—and each spoke different Cherokee dialects and were somewhat socially and politically independent [44, 45]. All of the Southeastern Cherokee spoke an Iroquois language that differed from the surrounding Muskogean, Siouan and Algic [46]. A series of wars with the Chicksaws and Creeks, British army (Montgomery, Grant), U.S. Revolutionary army (Rutherford) and Tennessee frontiersmen (Sevier) devastated the Cherokee settlements, with most settlements attacked and burned at least once during the mid- to late-1700s [47–49]. Many of the settlements were rebuilt and re-occupied [12, 28] until the Cherokee were forcibly removed to Oklahoma by the U.S. Army under the orders U.S. President Andrew Jackson in 1838.

I located the settlement sites using military maps, historical accounts, archeological research and surveys, historical markers and remaining mounds [12, 37, 40, 47, 48, 50–52]. I confirmed the locations with James (Johi) Grifin of the EBCI Tribal Historic Preservation Office and Ben Steere, Assistant Professor of Anthropology at Western Carolina University. GPS information is omitted to avoid revealing sensitive archeological locations.

### Distribution Surveys

Given the potential for spurious correlations in investigating historical patterning, I used three independent approaches in three Cherokee settlement areas to determine any linkage between current *G*. *triacanthos* distributions and a Cherokee cultivation legacy. First, I conducted an extensive 2012 ‘landscape survey’ for *G*. *triacanthos* trees following broad-scale transects that intersected with former settlements to avoid bias toward putative planting areas. Second, I conducted a 2014 ‘paired survey’ that specifically targeted Cherokee settlement locations and then, as a control, the nearest, generally adjacent, post-Cherokee settlement (i.e., European settlement areas typically founded around the time of the Cherokee removal in the mid-to-late 1800s). Finally, I used an ‘ATBI survey’ (All Taxa Biodiversity Inventory, http://www.dlia.org/atbi) on *G*. *triacanthos* locations in the Great Smoky Mountains National Park and examined proximity to nearest Cherokee settlements (versus control sites).

### Distribution Surveys—Landscape

Open and edge habitats throughout the Little Tennessee and Tuckaseegee watersheds (southern Appalachian Mountains, North Carolina, U.S.) were surveyed for *G*. *triacanthos* adults, saplings and seedlings in May 2012. This geographic area contains what are known as the Cherokee Middle Towns, as they are located between the higher-elevation, more northern Overhill Towns and lower, more southern Valley Towns. Initial surveys were conducted along historical Cherokee roadways that connected historical villages and towns. For example, the route that is now roughly US 441 and NC 28 was used by William Bartram to visit several Middle Towns, and it was later used by U.S. General Griffith Rutherford (“Rutherford Trace”) to attack at least a dozen Middle Towns in the late 1700s. These surveys were conducted along ~ 150 km of roadways at 1-km intervals, except in more intensively sampled stretches along Rutherford Trace, where surveys were conducted at 100-m intervals. Spotters looked for *G*. *triacanthos* along the drives, and then got out and walked and searched for 10-minute timed searches at the interval points. In addition, ~ 36 km of Tennessee and Cullasaja River shorelines were sampled with spotters in kayaks along each shoreline to search for adult trees in the floodplain or seedlings along the shoreline.

Where *G*. *triacanthos* was found, a team of surveyors was employed to survey the area for saplings and seedlings. Based on *a priori* observations and published reports of *G*. *triacanthos* shade intolerance (e.g., [21]), surveys were not conducted within forest interiors, which form the majority of cover in the study area [53]. Conversely, open habitat in the southern Appalachian region generally is maintained by anthropogenic activities such as farming, haying and mowing. As such, surveys were conducted *ad hoc* by site, the length determined by site size, and generally in haphazard patterns following buffer areas between forest edges and open areas.

GPS coordinates were recorded at each *G*. *triacanthos* found, and DBH was measured. Trees ≤ 1 cm DBH were categorized as seedlings, > 1 cm and ≤ 5 cm saplings and > 5 cm adults. For saplings and seedlings, the distance to the nearest putative parent tree (dbh > 25 cm) was measured. Pairing seedlings/saplings with parent trees generally was very obvious as the mature trees often occurred alone with the progeny clustered beneath them, but this study did not track seeds to germination, so parentage was not verified. For that reason, distances only were recorded for seedlings and sapling < 60 m from a putative parent (hence, truncating long-distance dispersal assessment). Observers also recorded habitat type (e.g., floodplain) and habitat use (e.g., cattle grazing). A random subset of trees across sites (*n* = 10) was selected to measure *G*. *triacanthos* "drip lines" (the distance between the trunk and furthest extension of branches—beyond which active seed dispersal is required to move beyond passive gravitation dispersal).

### Distribution Surveys—Paired

The 2012 landscape survey was exploratory, and hence many of the absences included elevations where *G*. *triacanthos* would not occur, and many presences were recorded in floodplains where the Cherokee often settled. The mean (±SE) elevation for non-Cherokee sites was 653±3 m whereas Cherokee sites were at 601±1 m, and 33% of the non-Cherokee sites were in floodplains whereas 66% of the Cherokee sites were floodplains. A second survey effort was employed in 2014 using a different approach to minimize search bias. Instead of exploring transects for *G*. *triacanthos*, three Cherokee Valley Town sites near Murphy, Hayesville and Brasstown, NC (U.S.), and two Overhill Town sites in the Great Smoky Mountains National Park (Cades Cove, Oconaluftee) were surveyed. For comparison, 'paired' surveys were conducted in post-Cherokee settled open habitats in Murphy, Hayesville and Brasstown adjacent (within 0.5 km) of the Cherokee sites. In the Great Smoky Mountains National Park, which was too heavily forested to use the immediately adjacent landscape for realistic controls, the closest post-Cherokee open habitats with similar elevation were used (e.g., Sugarlands). The mean (±SE) elevation for non-Cherokee sites in the control survey was 501±4 m whereas Cherokee sites were at 545±1 m, and 76% of the non-Cherokee sites were in floodplains whereas 24% of the Cherokee sites were floodplains. Trails and roadways for sampling (eight sites, 21.5 km total) were selected *a priori*, and intensive sampling was conducted by spotters walking along trails and roadways. When *G*. *triacanthos* was found, the same protocol as used for the landscape survey was used. Five-minute timed searches also were conducted at 100-m intervals to account for unoccupied habitat.

### Distribution Surveys—ATBI

The patterns found in the first two surveys were verified using an independent data set, the All Taxa Biodiversity Inventory (ATBI, http://www.dlia.org/atbi) collected in the Great Smoky Mountains National Park. Each park location that contained > 1 *G*. *triacanthos* was located, and then a control site was chosen in the nearest adjacent watershed at the same elevation, generally moving southward for locations on the western side of the park and eastward for locations on the southern side. After the *G*. *triacanthos* (*n* = 20) and control sites (*n* = 20) were chosen, Cherokee settlement sites then were researched and located. The distance between the *G*. *triacanthos* and control sites and the Cherokee settlements (not necessarily within the park boundaries) was measured.

### Data Analysis

The experimental treatment effects on *G*. *triacanthos* germination were evaluated using linear mixed model (LMM) analysis of variance (ANOVA) with shade and watering as fixed effects and experimental block as a random effect. The inclusion of a random block term for each cluster (*n* = 4) of seed trays accounted for unintended variation in germination response attributable to the environment rather than treatment effects. The LMM was evaluated using the (*lme4*) package in the R statistical program. The fixed and random effects were evaluated as an entire term two-way ANOVA using the (*LMERConvenienceFunctions*) package.

Recruitment success in the seed addition field experiment was analyzed using generalized linear mixed models (GLMMs) with transect (*n* = 6) as a random effect and soil moisture, diffuse light, temperature and vegetation cover as fixed effects. By including transect as a random effect, the model assumed that error within transects was not independent. Germination success (surviving seeds 2009) and survival (surviving plants 2011) were calculated as proportions assuming binomial error distributions. The GLMMs were evaluated based on the inclusion or exclusion of the fixed effects and interaction terms, and model selection was based on the Akaike information criterion (AIC). Potential collinearity between predictor variables was evaluated using the (*car*) package. The variance inflation factors for the predictor variables in all models were < 3, indicating they independently predict variance, and the binomial-distributed data were not over-dispersed (ϕ < 2). Because statistical analysis indicated that vegetation cover was a significant component controlling *G*. *triacanthos* demography, quantile regression was used to evaluate seedling survival (2009 to 2011) as a function of vegetation cover using the (*quantreg*) package for R. Quantile slope coefficient significance was evaluated using Markov chain marginal bootstrapping.

A *t* test was used to examine the mean difference in distance to Cherokee Middle Town archeological sites between plots that were occupied by *G*. *triacanthos* and plots that were unoccupied. A negative exponential model (y = e^(a−bx)^) was fit to the decay in *G*. *triacanthos* abundance with distance from Cherokee archeological sites using the (*nls*) package. Similarly, a negative exponential model was fit to seedling and sapling abundance per distance to parent trees. Survey transect (*n* = 22) was used as a random effect in a GLMM to evaluate dispersal distance as a function of grazing, floodplain and a grazing x floodplain interaction term assuming a Poisson distribution. A spatial map showing the proximity between *G*. *triacanthos* individuals and former Cherokee settlements was generated using the (*ggmap*) package. *t* tests also were used to examine mean difference in *G*. *triacanthos* trees between Valley and Overhill Cherokee settlement sites and adjacent control sites, and the mean difference in distance to nearest Cherokee archeological site between *G*. *triacanthos* and control sites in the Great Smoky Mountains National Park.

## Results

### Recruitment Niche—Greenhouse Studies

Germination success was much higher in scarified seeds (95.8%) than non-scarified seeds (5.6%). For scarified seeds, germination decreased significantly with shading (98.3% to 89.3%; *SS* = 0.327, *F* = 5.115, *p* = 0.025) but was unaffected by watering (*SS* = 0.028, *F* = 0.435, *p* = 0.511). There was no interaction between treatments (*SS* = 0.007, *F* = 0.102, *p* = 0.750).

### Recruitment Niche—Field Studies

**Fig 1:**
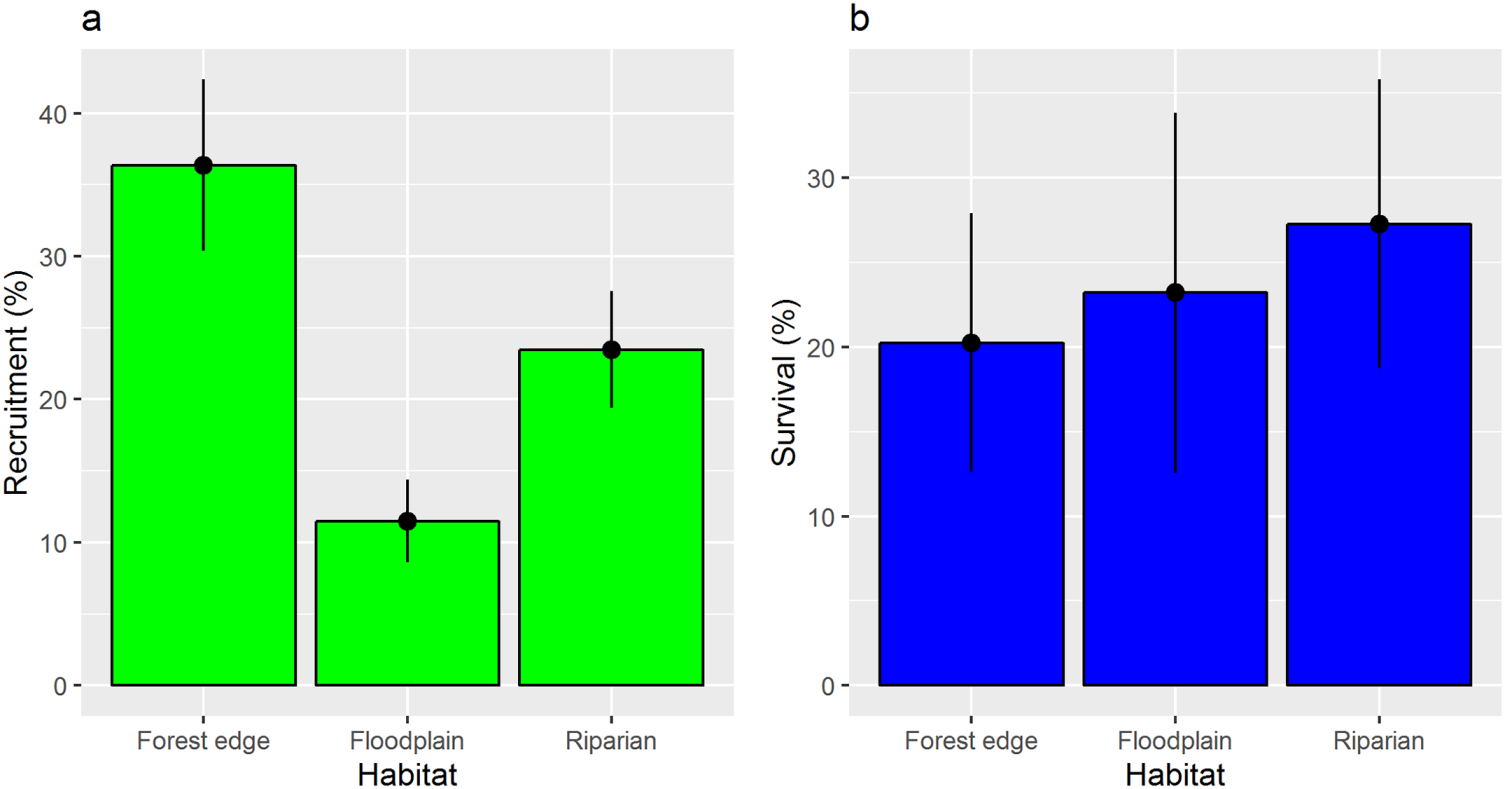
*Gleditsia triacanthos* (a) recruitment and (b) survival (mean ±SE) in forest edge, floodplain and riparian habitats pooled from two sites, Gibson Bottoms and Tessentee Bottomland Preserve in the Southern Appalachian Mountain region.

Of 630 planted *G*. *triacanthos* seeds in the field experiments, ~22% (138) germinated successfully and ~10% (60) survived the initial growing season. Seedling recruitment was highest in forest edge habitat and lowest in floodplain (Fig 1a, *SS* = 30.207, *F* = 8.184, *p* < 0.001), but post-establishment survival was the same across habitats (Fig 1b, *SS* = 0.435, *F* = 0.442, *p* = 0.644).

**Fig 2:**
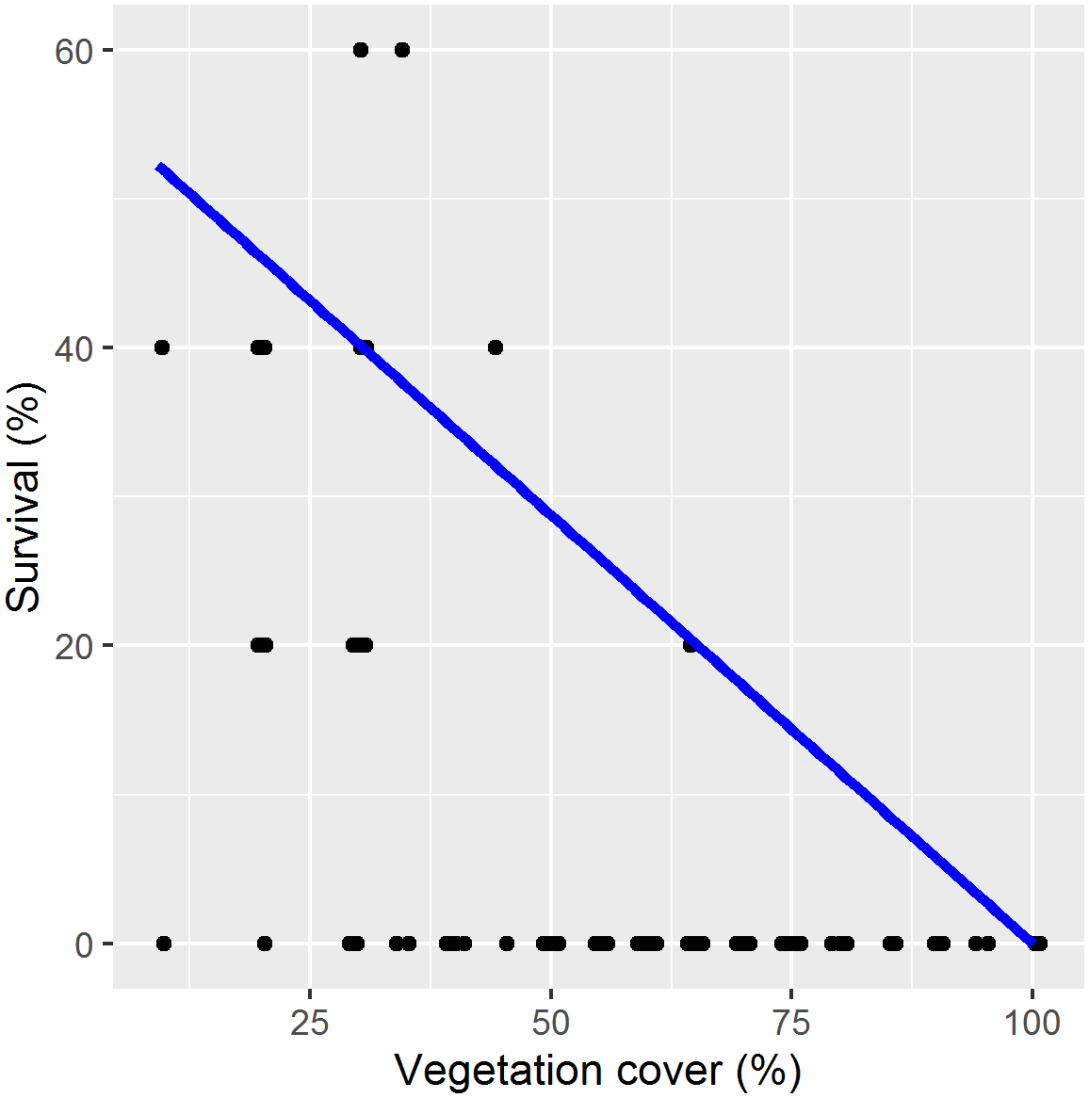
Maximum *Gleditsia triacanthos* seedling survival (%) as it was limited at the 95^th^ quartile by vegetation cover (%).

Across habitats, the best-fit model predicting recruitment success included vegetation cover and diffuse light. *Gleditsia triacanthos* seedling recruitment decreased significantly with increased vegetation cover (*coeff*. = -0.027, *SE* = 0.007, *z* = -4.243, *p* < 0.001), but the effect of diffuse light was not significant (*coeff*. = -0.002, *SE* = 0.006, *z* = -0.411, *p* = 0.681) and there was no interaction effect. The best-fit model for survival included vegetation cover and temperature. A significant cover x temperature interaction term indicated that *G*. *triacanthos* saplings did poorly where vegetation cover was higher and were little affected by temperature, but they did significantly worse where there was high vegetation cover and high temperatures (*coeff*. = -0.004, *SE* = 0.002, *z* = -1.984, *p* = 0.047). Quantile regression indicated that maximum *G*. *triacanthos* tree survival was significantly limited by vegetation cover (*coeff*. = -0.006, *SE* = 0.001, *t* = -4.059, *p* < 0.001), and almost no tree plantings survived where cover > 50% (Fig 2).

The environmental variables were essentially the same between the Gibson Bottoms and Tessentee sites, but not among the three study habitats within each site (S1 Table). The forest habitats were upland relative to the floodplain and riparian areas, with low light, moderate soil moisture and little herbaceous cover. The floodplain habitats were between the forested and riparian sites, and included the highest soil moisture and moderate light. The riparian habitats were immediately adjacent to the Little Tennessee River, but they were the least moist and had the highest light. The riparian sites had far greater herbaceous cover than the forest edge.

### Distribution Surveys—Landscape

A total of 1,076 trees were sampled (221 adults, 408 saplings, 447 seedlings). Mean (±SE) adult dbh was 16.8±0.9 cm. The trees generally were found at the edges of open habitats between 465–699 m elevation (sampling occurred between 320–1136 m).

**Fig 3:**
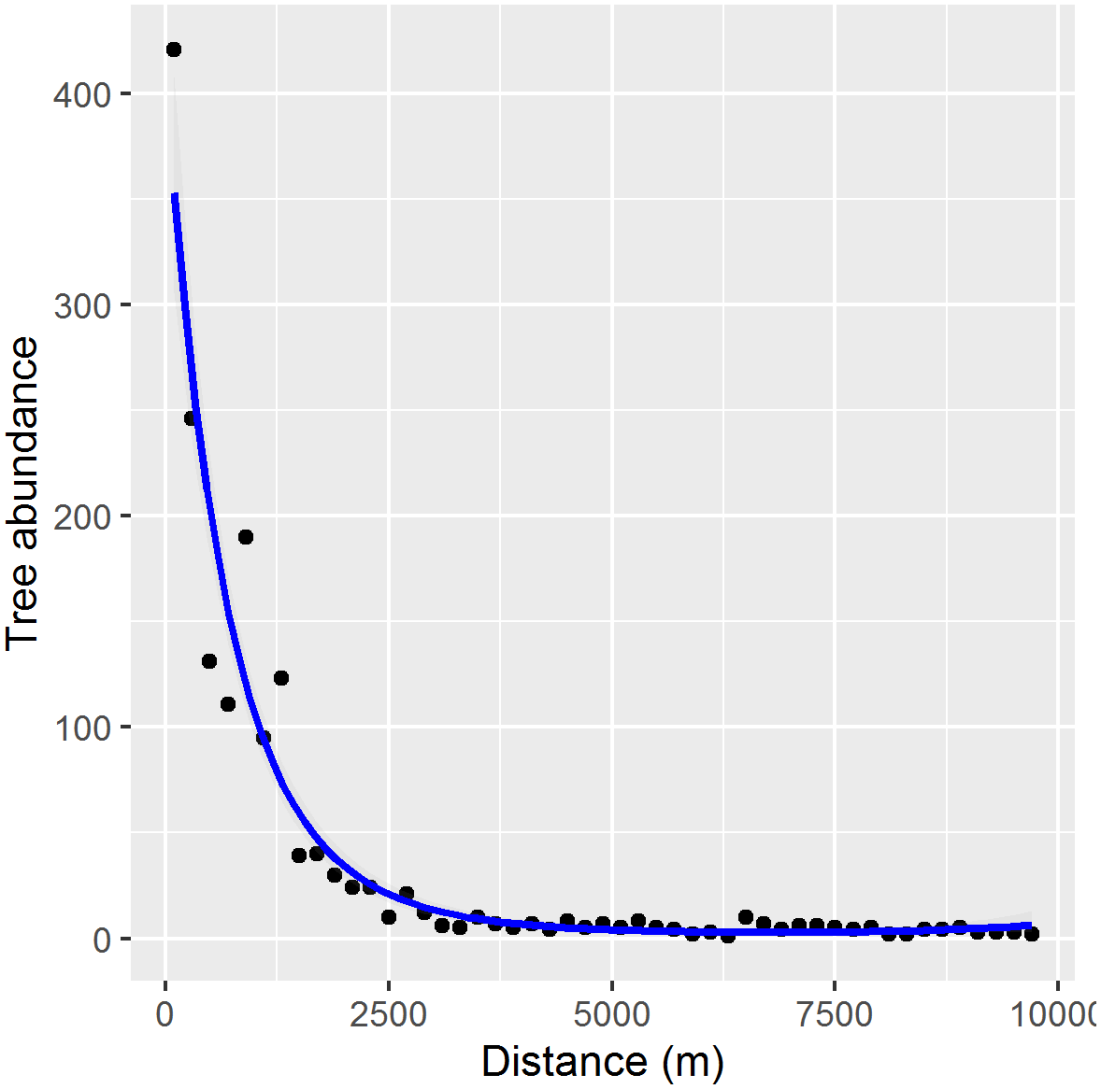
*Gleditsia triacanthos* tree abundance with increased distance from Cherokee settlement sites. The fitted line follows a negative exponential pattern.

The surveys indicated an association between *G*. *triacanthos* presence and abundance and former Cherokee settlements. *Gleditsia triacanthos* occupied habitat significantly closer to Cherokee sites than unoccupied habitat (mean occupied distance = 412 m, mean unoccupied distance = 3123 m; *t* = -22.806, *df* = 462, *p* < 0.001), and *G*. *triacanthos* tree abundance declined significantly with distance to Cherokee sites in a negative exponential pattern (*coeff*.*a* = 6.00, *SE* = 0.071, *t-value* = 84.672, *p* < 0.001; *coeff*.*b* = -0.002, *SE* = 0.0002, *t-value* = -10.115, *p* < 0.001) [Fig 3].

**Fig 4:**
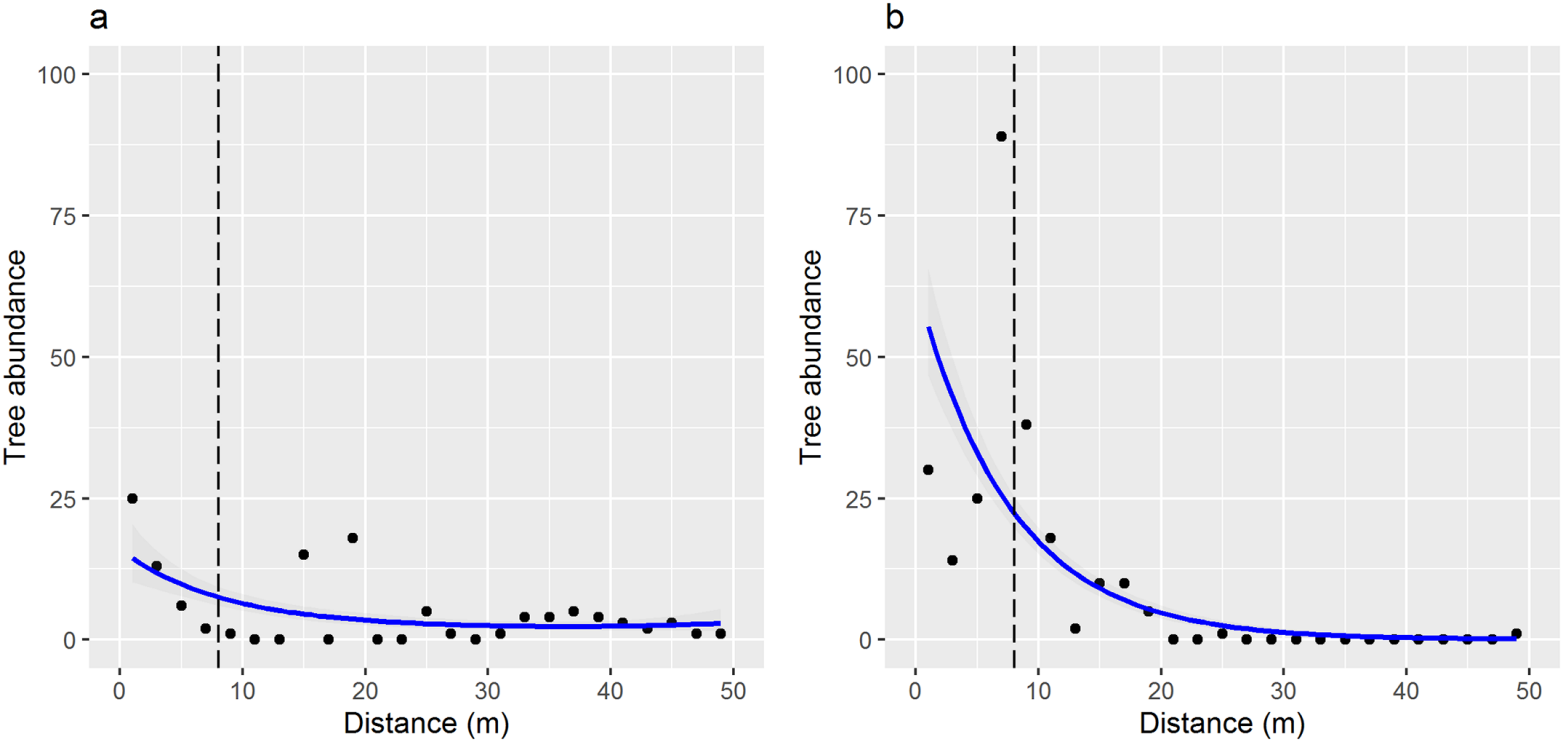
*Gleditsia triacanthos* seedling abundance with increased distance from mature parent trees in (a) grazed and (b) all other habitats. The fitted lines follow a negative exponential pattern. The dashed line in the graphic represents the tree drip line, which is the distance between the trunk and furthest extension of branches. The drip line represents an approximate maximum dispersal distance for passively dispersed seed pods that drop from the tree.

*Gleditsia triacanthos* seedlings appeared dispersal limited in all habitats, but moved much further from parent trees with cattle grazing, and appeared unaffected by waterflow. *Gleditsia triacanthos* seedling/sapling dispersal distance increased significantly in grazed habitats (*coeff*. = 0.821, *SE* = 0.060, *z* = 13.718, *p* < 0.001) but was not influenced by floodplains (*coeff*. = 0.042, *SE* = 0.123, *z* = 0.349, *p* = 0.727). Mean (±SE) distance in grazed habitats (17.6±0.8 m) was twice as far as floodplains (8.3±0.2 m). In grazed habitats, seedling and sapling abundance declined significantly with distance to parent tree in a negative exponential pattern (*coeff*.*a* = 3.520, *SE* = 0.289, *t-value* = 12.311, *p* < 0.001; *coeff*.*b* = -0.317, *SE* = 0.124, *t-value* = -2.567, *p* < 0.001) [Fig 4a]. Dispersal distances declined in a similar negative exponential pattern in ungrazed floodplains (*coeff*.*a* = 4.282, *SE* = 0.277, *t-value* = 15.451, *p* < 0.001; *coeff*.*b* = -0.075, *SE* = 0.030, *t-value* = -2.531, *p* < 0.001) [Fig 4b]; however seedling/sapling abundance peaked at approximately 8 m, which fell within the mean (±SE) drip line for mature trees 8.3±0.6 m, indicating that the seeds fell beneath the trees and germinated.

### Distribution Surveys—Paired and ATBI

The 2014 survey of Cherokee and post-Cherokee settlements indicated that *G*. *triacanthos* trees occurred at significantly greater densities at former Cherokee sites than nearby control sites (*Cherokee*: 0.47 trees km^-2^, *control*: 0.01 trees km^-2^; *t* = 3.420, *df* = 4.029, *p-value* = 0.027) [S1a Fig]. Similarly, the ATBI data from the Great Smoky Mountains National Park indicated that *G*. *triacanthos* trees occurred significantly closer to former Cherokee sites than nearby control sites (*Cherokee*: 2.0 km, *control*: 4.1 km; *t* = -2.559, *df* = 13.99, *p-value* = 0.023) [S1b Fig].

## Discussion

Plants populations can persist in suboptimal habitat for extended periods [2, 8] and occur in disequilibrium with suitable habitat at large scales [9]. The distribution of *G*. *triacanthos* trees in the southern Appalachian Mountain region appears as much, if not more, a function of Cherokee facilitation centuries ago as does the tree's own habitat limitations. Moreover, it appears severely dispersal limited without extinct megafauna or anthropogenic cultivation, except where transported within pastures by domestic cattle.

*Gleditsia*. *triacanthos* recruitment and survival were most limited by vegetation cover, and unaffected (even recruitment limited) by increased soil moisture in the seed addition experiment. The field plantings suggested that mesic bottomland habitat was the least suitable for *G*. *triacanthos* recruitment, despite that the tree often is found in bottomlands and mesic habitats [20–23]. Once *G*. *triacanthos* reached the sapling stage in the seed introductions, however, survival appeared unaffected by habitat type, consistent with other findings that adult *G*. *triacanthos* trees can persist in marginal habitat [21]. Overall, the published literature and results presented here indicate a consistent pattern that *G*. *triacanthos* is limited by shade/vegetation cover but not moisture. Hence, the *G*. *triacanthos* association with Cherokee settlements in the Southern Appalachian Mountain region did not appear a function of the Cherokee affinity for riverine habitat, but was more likely because the Cherokee cultivated the trees and/or they disturbed the vegetation in these habitats.

Cherokee in the Qualla phase (A.D. 1450–1838) occupied alluvial bottomlands in the Southern Appalachians, only using the higher mountain areas for occasional or seasonal hunting and gathering [19, 52, 54]. The Cherokee in this phase practiced hunting and gathering, but their food economy primarily was agricultural [19, 52, 54, 55]. The Cherokee grew the common triad of Native American crops—corn, beans, squash—along with additional cultivars such as chenopod and “little barley” [40, 55, 56]. The Cherokee also cultivated woody plants, as evidenced by the widespread appearance of peach trees after European contact [56, 60], and the Cherokee had three locations in the Southeastern U.S. named "honey locust place" believed to be orchards [28]. Still, it remains possible that the positioning of *G*. *triacanthos* groves near Cherokee settlements could be unintentional with trees sprouting up from seeds scarified during food processing and discarded near settlements or in middens.

Acorns and hickory nuts also were common food items, along with *G*. *triacanthos*, but they could have been gathered, and the tree presence near settlements could be the result of land use practices, such as burning [17, 19, 40, 57–59]. The appearance of weedy plant species with Cherokee settlement suggests that land clearing was common [54, 55], which also would favor *G*. *triacanthos*. Vegetation cover consistently interfered with *G*. *triacanthos* regeneration in the greenhouse and field experiments, verifying that *G*. *triacanthos* requires open, possibly disturbed, habitat. Vegetation disturbance is associated with agriculture, terraforming and other anthropogenic-driven activities [18] as well as Native American burning [19]. Given *G*. *triacanthos* success in drier, open habitats, the tree should be plentiful around Southern Appalachian pastures, forest edges and roadways [which are increasing in the region, 53], but it is not—only occurred in those habitats near Cherokee settlements and not near European settlements. Unoccupied suitable habitat suggests dispersal limitation [2, 61], and the flexibility exhibited by *G*. *triacanthos* in experimental translocations across habitats suggests that the tree could inhabit much more Southern Appalachian habitat with greater dispersal.

The disappearance of large mammals (e.g., mastodons) at the end of the Pleistocene (13,000–10,000 ya) also meant the loss of facilitative dispersers capable of ingesting and spreading the seed-bearing propagules of many large-fruited trees in North and South America [30, 62, 63]. With their key dispersers gone, the fruits of trees such as *G*. *triacanthos* L. were left to accumulate beneath parent trees—subject to heavy predation by insects and rodents [21] and lacking the seed coat scarification required for standard germination [64, 65]. Estimates for the degree of overlap between colonizing humans and megafauna in North America vary from 0 to 1000 years [66, 67]. However, several millennia occurred before agriculture appeared [19, 68] so a long gap likely occurred between megafaunal extinction and potential human dispersal of *Gleditsia triacanthos*. As such, the distribution of persisting large-seeded species likely shrank considerably into small, remnant populations [30, 69]. For *G*. *triacanthos*, remnant populations likely were concentrated in the Midwestern U.S [21, 70].

Smaller wildlife such as ungulates, birds and small mammals can act as surrogate *G*. *triacanthos* disperses [21, 30, 33, 71], and *Odocoileus virginiana* (white-tailed deer) may occasionally provide long-distance seed dispersal [72, 73]. However, *O*. *virginianus* populations in the southern Appalachian Mountain region are among the lowest in the eastern U.S. [74], and the results presented here, and elsewhere [23, 33], suggest very limited dispersal by any means as *G*. *triacanthos* seedlings and saplings occur very near or immediately below parent trees—except where cattle graze.

Colonial and post-colonial livestock possibly acted as surrogate *G*. *triacanthos* dispersers [21, 30, 33] with the demise of Cherokee cultivation. However, pasturelands in the southern Appalachian Mountains are not as expansive as those in the Western U.S., so livestock dispersal is truncated by fences. Cattle likely eat the *G*. *triacanthos* pods and disperse the seeds through manure [21, 30, 33]. Cattle also might graze on the *G*. *triacanthos* seedlings, which could cause a distance dispersal pattern if herbivory was concentrated below parent trees where the seedlings were most abundant. However, seedlings were found at much greater distances in grazed than in ungrazed habitat, but the proportion of seedlings (seedlings sample^-1^) differed little between grazed (27.3%) and ungrazed (24.8%) habitats, giving no hint of seedling mortality by cattle.

Finally, waterflow has been proffered as a surrogate *G*. *triacanthos* dispersal mechanism in the absence of extinct megafauna [15, 16] but in the Southern Appalachian region extensive riparian and floodplain surveys revealed no evidence of shoreline transport or recruitment. Most importantly, dispersal distance in floodplains did not differ from other habitats, and it was half the distance of that found in grazed habitat.

*Gleditsia triacanthos* appeared to be a very important plant for the Cherokee, but they were not the only Native Americans who used the tree. The Cherokee largely occupied settlements with Muskogean rather than Cherokee/Iroquois names, suggesting they took over those sites sometime before the Qualla phase. Moreover, during the Qualla phase, the Cherokee culture became less distinct from that of other Southeastern cultures suggesting that they adopted regional ideas [37–40]. Hence, the cultivation of *G*. *triacanthos* by the Cherokee could have been inherited or learned from other nations. Along with Cherokee, there are reports that the Creeks used *G*. *triacanthos* as medicine [26, 36, 75, 76], as did several tribes north of the Cherokee [77–79]. For the Cherokee, however, *G*. *triacanthos* was more than medicine as the pods also served as a food item, the wood was preferred for game sticks and the tree itself was a spiritual icon [27, 28].

A Cherokee cultivation legacy persists to the extent that *G*. *triacanthos* distributions in the southern Appalachian region appear more strongly patterned by centuries-old settlement preferences than its own niche requirements. Native Americans likely transported numerous native plant species throughout the Eastern United States, and indigenous imprints on modern species distributions are widespread [17, 19, 58, 80]. As such, pre-European activity may be underrated as a factor influencing modern species distributions.

These results also suggest that great caution must be exercised when correlating species distributions with current climate to assume niche requirements [*see* 22]. Indeed, predictive models may be better parameterized with pre-settlement land surveys than putative niche requirements [58]. Suitable habitat often is defined by abiotic drivers (e.g., temperature and moisture), and the approach often works well in predicting species distributions at large scales [81]. For species dependent on biotic interactions, however, suitable habitat may better be defined by the presence of facilitators or mutualist partners [5–7]. *Gleditsia triacanthos* appears to have lost two dispersal agents in extinct Pleistocene megafauna and Cherokee cultivation. Given that *G*. *triacanthos* can be invasive on other continents, it seems to easily form new dispersal partnerships, and it commonly is used as an urban tree throughout the Midwestern and Northeastern U.S., suggesting a modern anthropogenic disperser. In each case, the niche requirements of the disperser, be it modern or historical, best predicts the distribution of the dispersed.

## Supporting Information

S1 Fig Second surveys. Barplots showing *Gleditsia triacanthos* abundance in Cherokee and control sites from 2014 sampling and *G*. *triacanthos* distance from Cherokee and control sites using data from the All Taxa Biodiversity Inventory.
(PDF)

S1 Table Site characteristics. Environmental variables across sites and habitat types. (PDF)
