# Supplementary material for: Ghosts of Cultivation Past - Native American Dispersal Legacy Persists in Tree Distribution
Source: PLoS One. 2016 Mar 16;11(3):e0150707. doi: 10.1371/journal.pone.0150707 (PMC4794212; doi:10.1371/journal.pone.0150707)

S2 Figure. Barplots showing *Gleditsia triacanthos* (a) abundance in Cherokee and control sites from 2014 sampling (b) and *G. triacanthos* distance from Cherokee and control sites using data from the All Taxa Biodiversity Inventory.

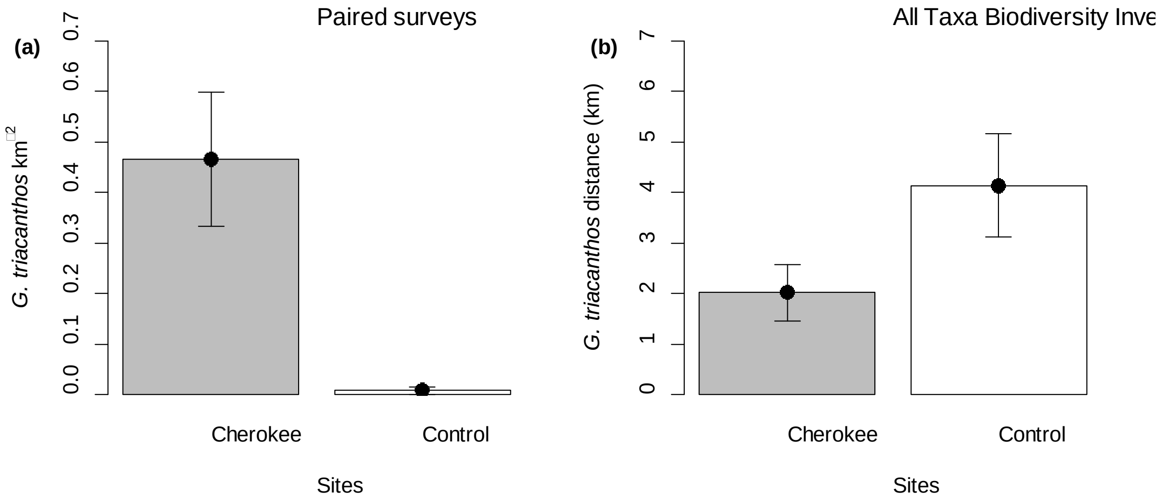

Supplement: S1 Fig — Barplots showing Gleditsia triacanthos abundance in Cherokee and control sites from 2014 sampling and G. triacanthos distance from Cherokee and control sites using data from the All Taxa Biodiversity Inventory. (PDF) [file pone.0150707.s001.pdf]
