# Supplementary material for: Ghosts of Cultivation Past - Native American Dispersal Legacy Persists in Tree Distribution
Source: PLoS One. 2016 Mar 16;11(3):e0150707. doi: 10.1371/journal.pone.0150707 (PMC4794212; doi:10.1371/journal.pone.0150707)
Supplement: S1 Table — (PDF) [file pone.0150707.s002.pdf]

S1 Table. Environmental variables (mean ± SD) across sites and habitat types. The environmental variables are diffuse light (light), soil moisture (moist), ground temperature (temp) and vegetative ground cover (cvr) at two sites, Gibson Bottoms (Gibson) and Tessentee Bottomland Preserve (Tessentee) and three habitat types, forest, wetland and riparian.

	Light (%)	Moist (%)	Temp (°C)	Cvr (%)
Gibson	36.2 ±23.8	10.6±5.7	20.2 ±0.2	62.5 ±27.0
Tessentee	27.1 ±20.2	13.7±3.1	19.6 ±0.7	59.4 ±14.0
Forest edge	13.1 ±11.0	11.6 ±3.9	19.8 ±0.5	34.3 ±17.3
Floodplain	29.9 ±15.9	16.6 ±3.0	19.3 ±0.5	71.1 ±14.1
Terrace	49.9 ±21.6	8.3 ±2.6	20.5 ±0.3	68.5 ±14.7
